# A Three-Dimensional Model of Bacterial Biofilms and Its Use in Antimicrobial Susceptibility Testing

**DOI:** 10.3390/microorganisms12010203

**Published:** 2024-01-19

**Authors:** Hala R. Ali, Pamela Collier, Roger Bayston

**Affiliations:** 1 Unit of Injury, Inflammation and Recovery Sciences, Queen’s Medical Centre, School of Medicine, University of Nottingham, Derby Road, Nottingham NG7 2UH, UK; alihala312@gmail.com; 2 Bacteriology Department, Animal Health Research Institute (AHRI), Agriculture Research Centre (ARC), Dokki, Giza 12618, Egypt; 3 Division of Cancer and Stem Cells, Queen’s Medical Centre, School of Medicine, University of Nottingham, Derby Road, Nottingham NG7 2UH, UK; pamela.collier@nottingham.ac.uk

**Keywords:** 3D biofilm model, combination therapy, chronic wound infection, *S. aureus* biofilm, antimicrobial susceptibility testing

## Abstract

(1) Background: The discrepant antimicrobial susceptibility between planktonic and biofilm bacterial modes poses a problem for clinical microbiology laboratories and necessitates a relevant 3D experimental model allowing bacteria to grow in biofilm mode, in vitro, for use in anti-biofilm susceptibility testing. (2) Methods: This work develops a 3D biofilm model consisting of alginate beads containing *S. aureus* biofilm and encased within two thick layers of alginate matrix. The constructed model was placed on a thin Boyden chamber insert suspended on a 24-well culture plate containing the culture medium. The antibacterial activity of bacitracin and chlorhexidine digluconate (CD), either combined or separately, against 2D *S. aureus* culture was compared to that in the 3D biofilm model. Quantitative analysis and imaging analysis were performed by assessing the bacterial load within the matrix as well as measuring the optical density of the culture medium nourishing the matrix. (3) Results: The 3D biofilm model represented the typical complex characteristics of biofilm with greater insusceptibility to the tested antimicrobials than the 2D culture. Only bacitracin and CD in combination at 100× the concentration found to be successful against 2D culture were able to completely eliminate the 3D biofilm matrix. (4) Conclusions: The 3D biofilm model, designed to be more clinically relevant, exhibits higher antimicrobial insusceptibility than the 2D culture, demonstrating that the model might be useful for testing and discovering new antimicrobial therapies. The data also support the view that combination therapy might be the optimal approach to combat biofilm infections.

## 1. Introduction

Clusters of bacterial cells resident in self-produced extracellular matrix, known as biofilm, are commonly involved in chronic and resistant wound infections, delaying or preventing healing [1,2]. Biofilm is a major contributor to delayed wound healing, as it provides protection against antimicrobial agents and host defense mechanisms [3,4]. Such chronic wounds, often affecting people with underlying pathologies such as diabetes or peripheral vascular insufficiency, are a great burden on patients and healthcare services. Bacteria growing in biofilm mode are up to 1000 times less susceptible to antimicrobials than their planktonic counterparts [5], and achieving such high concentrations is a challenge. However, the accessibility of chronic wounds enables consideration of topical antimicrobial therapy, by which such high concentrations might be achieved, and for which agents unsuitable for systemic use can be considered. The discrepant antimicrobial susceptibility between planktonic and biofilm modes poses a problem for clinical microbiology laboratories, as the bacteria are tested for susceptibility as either planktonic cultures (in liquid media) or as colonies on agar plates, and both these modes give a much lower MIC [6]. The two bacteria most commonly implicated in chronic wound contamination are *Pseudomonas aeruginosa* and *Staphylococcus aureus* [7].

The few methods available for determination of the concentration of antimicrobial that can kill most or all of biofilm bacteria are expensive, and while they yield useful research data, they take too long to be clinically useful. Therefore, a relevant experimental model is required that allows bacteria to grow in biofilm mode but is inexpensive and relatively rapid.

Previous in vitro models have employed an inorganic substrate (such as glass, plastic and metal) as a surface for biofilm formations. The solid surfaces used to enhance biofilm attachment in vitro allow larger biofilm accumulation. However, although they are uniformly produced and easy to use, it has been shown that biofilms formed on these non-biological surfaces are morphologically different from those grown in in vivo systems [8,9,10]. A study comparing the characteristics of in vitro biofilm with those in vivo demonstrated that in vivo biofilm clusters are numerous and physically smaller in diameter, and lack the mushroom-like structures reported in vitro [8]. Growth of biofilm in in vivo wound infections shares these distinct structural characteristics, as it is established deeper within a semi-solid matrix of tissue and exudate and does not need a surface for attachment.

Recently, researchers have observed the same phenomena in testing of anti-cancer drugs, which have been well established in microbiology for some time [11]. Traditionally, anti-cancer drugs were tested against cancer cells growing as monolayers, and it has been known for some time that the results of these assays are often not borne out in the patient. Current anticancer testing now uses what are termed 3D cell cultures, where the cancer cells are grown into “tumor” formation in a supportive gel matrix [12]. Microbiologists will appreciate the concordance with well-established principles regarding biofilms and antimicrobials. Thus, we developed a 3D model of *S. aureus* biofilms and evaluated its efficiency in testing the synergistic effect of two antimicrobials.

## 2. Materials and Methods

### 2.1. Development of 2D S. aureus Biofilm

**Figure 1:**
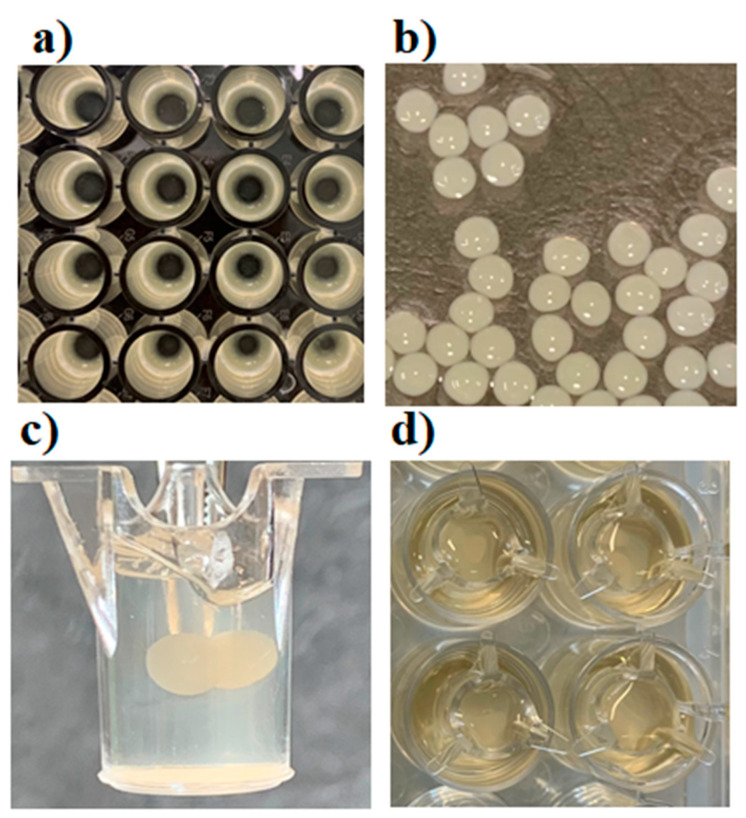
Construction of biofilm model: (**a**) *S. aureus* biofilm in 96-well plates; (**b**) *S. aureus* alginate beads; (**c**) biofilm beads embedded within alginate matrix in a 24-well Boyden chamber insert; and (**d**) Boyden chamber insert carrying the 3D biofilm model and hanging in LB medium. Images were captured using an Iphone XS camera.

*S. aureus* SH1000 expressing GFP was grown on Luria Bertani broth (LB; Gibco, Thermofisher Scientific, Leicester, UK), supplemented with chloramphenicol (25 µg/mL), overnight with shaking at 37 °C. Then, 100 µL of the bacterial culture was distributed in a 96-well microtitre plate. The microtitre plates were then wrapped with Parafilm and kept in a closed container accompanied by two 200 mL beakers full of water to maintain humidity. After incubation for 72 h at 37 °C in this condition, the planktonic cells were removed by inverting the 96-well plate over a waste tray and shaking it gently. Then, the biofilm layer was scraped and aspirated using a pipette (Figure 1a).

### 2.2. Synthesis of Biofilm Beads

Briefly, according to a published method [13] with slight modifications, 4% alginic acid sodium salt (Sigma Aldrich, St. Louis, MO, USA) was first prepared; then, 250 µL of fresh biofilm bacteria, collected as above, was mixed with 1.5 mL of 4% alginate. Using a sterile syringe and a 25 G needle, the biofilm/alginate drops were dispensed into a crosslinking solution of CaCl_2_ 1.5%/13 mM HEPES buffer (Sigma Aldrich) while gently swirling the beaker. The beads were allowed to crosslink at room temperature for 20 min. The crosslinking solution was then removed, and the beads were washed twice with 13 mM sterile HEPES buffer. The synthesized biofilm beads were distributed in 96 well plates containing LB broth (one bead per well). The stability of the beads was confirmed by keeping the beads in LB broth medium for 2 weeks and changing the medium every 3 days. The fabricated beads were imaged using confocal microscopy at different time points including day zero, day 5, and day 10. Beads kept in LB medium for 5 days to mature were used in the biofilm model.

### 2.3. Construction of 3D Biofilm Model

The 3D biofilm model was designed as two layers of alginate matrix encasing the biofilm beads between them. Briefly, the first layer of the matrix, consisting of 150 µL of 4% alginate, was placed on ThinCert^®^ Cell Culture with a 1.0 µm pore diameter and transparent sterile inserts for 24-well plates (Greiner Bio One, Gloucestershire, UK). The first matrix layer was cross-linked using CaCl_2_/HEPES buffer for 5 min, then washed twice with 13 mM sterile HEPES buffer. Two 5-day-old biofilm beads were placed on the first alginate layer, followed by covering with another layer of 4% alginate (150 µL). The constructed matrix consisted of two biofilm beads embedded between two layers of 4% alginate matrix (Figure 1c).

### 2.4. Testing Antimicrobials against Planktonic Bacteria

*S. aureus* SH1000 was tested against a combination of bacitracin (Sigma Aldrich) with chlorhexidine digluconate (20% solution, Sigma Aldrich) (CD) using a checkerboard assay as previously described [14]. Briefly, CD was serially diluted 2-fold in a 96-well plate from column 1 to 11, and bacitracin was serially diluted 2-fold from row A to G. Column 12 contained a serial dilution of bacitracin alone, while row H contained a serial dilution of chlorhexidine alone. The bacterial inoculum was previously prepared by growing one colony overnight in Luria Bertani broth, then adjusted to 0.1 optical density (OD) at 620 nm wavelength and diluted 1:10. This was distributed in the 96-well plate (100 µL/well). After 24 h incubation at 37 °C, the OD was measured using a plate reader (Agilent BioTek Synergy H1, Boston Industries, Walpole, MA, USA) at a wavelength of 630 nm. An OD reading below 0.1 was considered no growth while that ≥0.1 was considered growth. The fractional inhibitory concentration (FIC) index was calculated according to the following formulas: FIC bacitracin = MIC-bacitracin + CD/MIC-bacitracin, FIC-CD = MIC-CD + bacitracin/MIC-CD, FIC Index = FIC-bacitracin + FIC-CD. FIC Index values were then interpreted according to Mun et al. (2013) [15] and Si, Wei et al., 2018 [16]: synergy (FIC Index ≤ 0.5); partial synergy (FIC Index > 0.5 to ≤0.75); additivity (FIC Index > 0.75 to ≤1); no interaction (indifference) (FIC Index > 1 to ≤4); and antagonism (FIC Index > 4.0).

### 2.5. Evaluating the Constructed 3D Biofilm Model

The constructed biofilm model was treated with 1× and 100× of 6.25 µg/mL bacitracin and 0.000029% CD, either separately or combined, for 3 days. At the end of the incubation, the alginate matrix of treated and untreated controls was removed using a sterile loop from the Boyden chamber insert, then un-crosslinked by incubation in 1 mL of 33 mM trisodium citrate (Fisher Scientific, Hampton, NH, USA) for 20 min. Subsequently, the bacteria in the un-crosslinked matrix were quantified using 10-fold serial dilutions followed by plating on blood agar. The bacterial culture medium that contained the antimicrobial was also subjected to optical density measurements using a plate reader at a wavelength of 630 nm.

### 2.6. Confocal Microscope Imaging

The biofilm beads were collected from the constructed 3D biofilm model after the incubation. Subsequently, the beads were washed with PBS, then stained with 2 µM propidium iodide for 10 min in the dark at room temperature. After the incubation, the beads were washed with PBS twice and placed on a sample pack Cell ViewTM slide (Greiner Bio One, Gloucestershire, UK), then visualized using an LSM 710 confocal microscope with a 40× water objective (1.2 NA). GFP mode (444 nm excitation, green emission) in combination with PI mode (555 nm excitation, red emission) was used. Image analysis was conducted using Image FIJI-win 32.

### 2.7. Statistical Analysis

SPSS software version 22 was used to analyze the data using a one-way ANOVA test. *p* < 0.05 was considered statistically significant.

## 3. Results

### 3.1. Synthesis of Biofilm Beads

**Figure 2:**
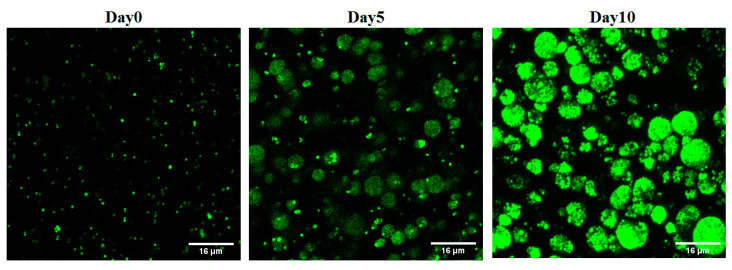
Confocal microscope images of *S. aureus* biofilm-laden beads at different time points. The number of biofilm clusters shown increased with time.

Different concentrations of sodium alginate (1–6%) were used to fabricate alginate beads, and the stability and stiffness of the beads were monitored. The 1–2% sodium alginate beads were found to deform easily with handling and degrade with incubation, while the 5% and 6% sodium alginate beads broke and cracked after two days of incubation. To fabricate biofilm beads with a core of maximum thickness, a 4% sodium alginate solution was used and large, uniform, and stable beads with an average diameter of 1.25 mm were successfully produced (Figure 1b). The imaging analysis of the 4% beads revealed that biofilm clusters increased in size and number with time (Figure 2).

### 3.2. Evaluating the Constructed 3D Biofilm Model

**Figure 3:**
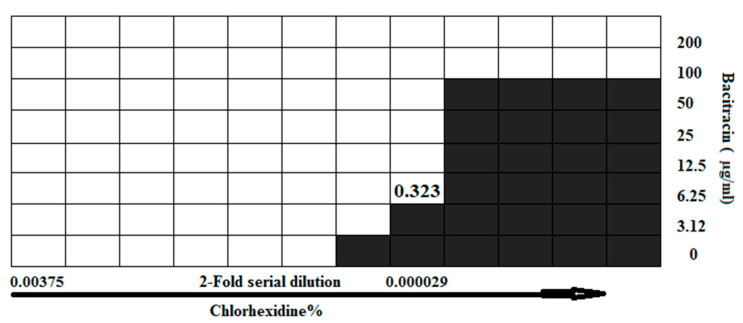
Checkerboard result of testing a combination of bacitracin with CD against planktonic *S. aureus* SH1000. Synergistic inhibition was observed with 0.323 FICI. The black area represent the bacterial growth with OD more than 0.1, while white area is considered no growth with OD below 0.1.

**Figure 4:**
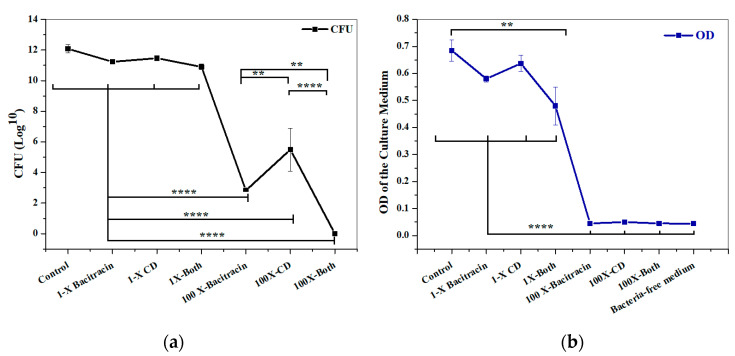
Response of 3D biofilm matrix model to treatment with 1× and 100× of chlorhexidine digluconate and bacitracin, either combined or separately. (**a**) Bacterial quantification (CFU/mL) and (**b**) OD of the culture medium. ****: *p* = ≤0.0001, **: *p* = ≤0.01.

A checkerboard assay was performed to test a combination of bacitracin with CD against 2D planktonic *S. aureus*. The result showed a significant synergistic interaction observed at a combination of 6.25 µg/mL bacitracin and 0.000029% CD, with a fractional inhibitory concentration index (FICI) of 0.323 (Figure 3). To evaluate the constructed 3D biofilm model, bacitracin and CD at 1× and 100× of the concentration that successfully produced significant synergy, as applied against planktonic bacteria, were applied on the 3D biofilm model either separately or combined. The efficiency of 3D biofilm model in antimicrobial susceptibility testing was evaluated by quantification of the bacterial load within the solid biofilm model, and by measuring the OD of culture medium in which the model was hung. The initial visual observation was the high turbidity of the culture medium of the biofilm models treated with 1× of treatments, and the same turbidity was also observed with the untreated model. In comparison, the culture medium of the models treated with 100× of treatments remained clear. As clarified in Figure 4, no significant difference in CFU was observed between the untreated control and 1× treatments, including 6.25 µg/mL bacitracin, 0.000029% CD, and both combined. In contrast, significant reductions in the CFU and OD of the medium were observed with 100× treatments (*p* = ≤0.0001). Bacterial load was also significantly decreased at 100× concentration with the bacitracin/CD combination compared to bacitracin (*p* = ≤0.0001) and CD alone (*p* = ≤0.01). CFU was also significantly reduced with 100× of bacitracin when compared to that with CD. However, the OD did not show any significant difference with 100× concentration of the combination or each separate treatment. A clear correlation was also noticed between CFU count and measurement of the OD of culture medium of control or 1× treatments. In contrast, no correlation was observed between the CFU and OD of 100× treatments. Most importantly, the combination tested at 100× of 6.25 µg/mL bacitracin and 0.000029% CD was the only treatment that cleared the biofilm from the model and the culture medium.

### 3.3. Imaging Result

**Figure 5:**
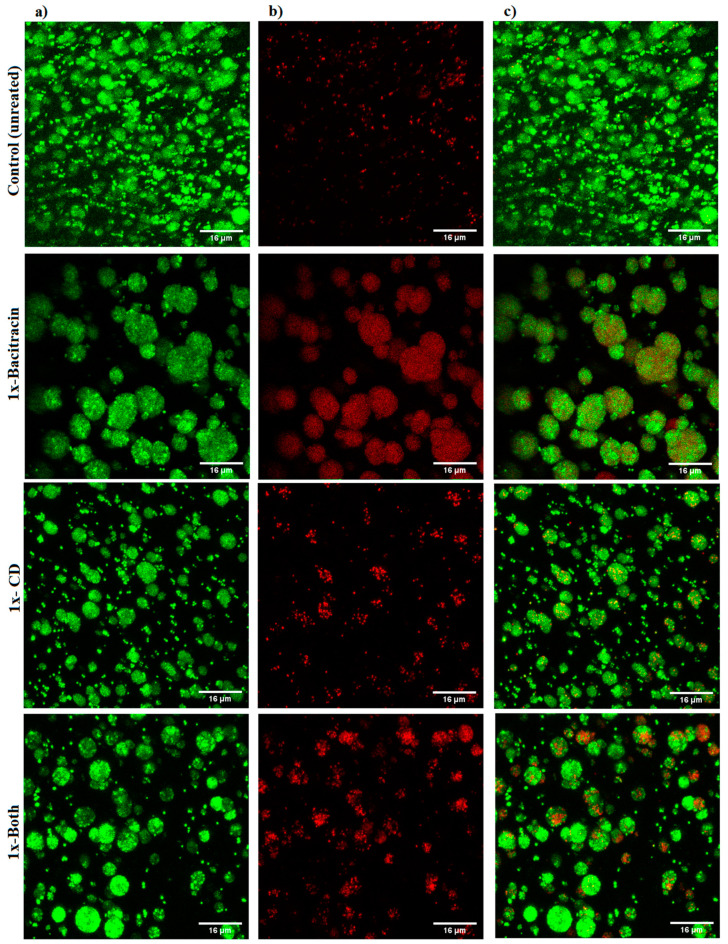
Confocal microscope images of *S. aureus* biofilm beads collected from 3D biofilm model treated with 1× bacitracin or chlorhexidine digluconate either combined or separately, versus untreated control. The biofilm beads embedded between two layers of alginate matrix were exposed to treatments for 3 days. (**a**) Green channel; (**b**) red channel; and (**c**) mixed channel.

**Figure 6:**
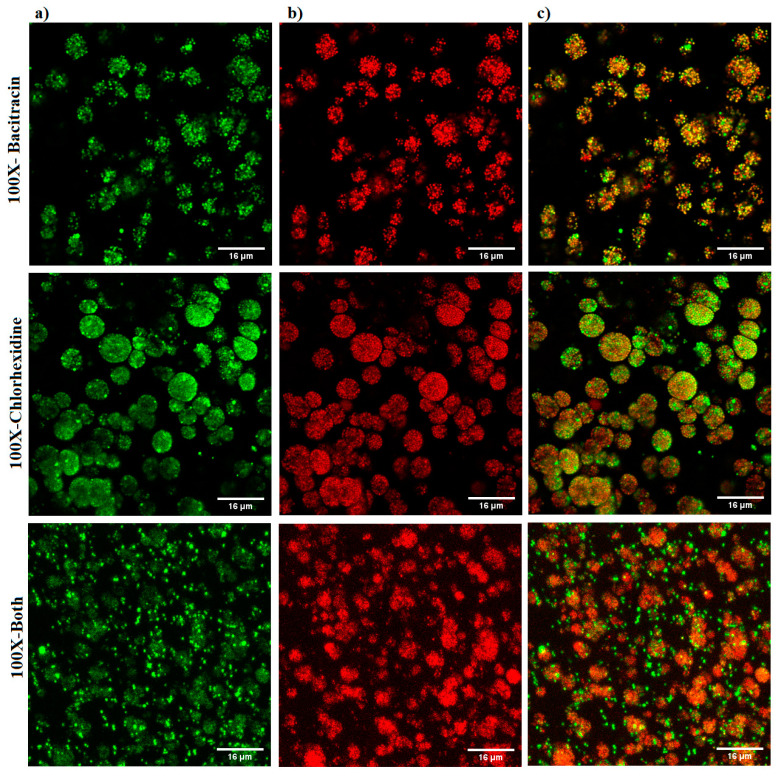
Confocal microscope images of *S. aureus* biofilm beads collected from a 3D biofilm model treated with 100× bacitracin or chlorhexidine digluconate either combined or separately. Dead bacterial cells lost GFP, and the PI signals increased. (**a**) Green channel; (**b**) red channel; and (**c**) mixed channel.

The qualitative imaging result was dependent on decline in GFP signals and increase in PI signals, as indicative of a reduction in cell viability and an increase in cell death. The results showed a slight increase in PI signals with 1× combination compared to each treatment alone and the untreated control (Figure 5). A marked increase in the intensity of PI signals was observed with the three 100× treatments, but the highest intensity of PI signals was demonstrated with the 100× combination (Figure 6).

## 4. Discussion

*S. aureus* resident in the biofilm matrix are found in chronic wound infections, with significantly reduced susceptibility to current remedies including disinfectants and antibiotic treatments. Chronicity has mainly been attributed to biofilm infection in several studies. These studies cited that up to 90% of resistant-to-healing wounds are infected with biofilms. However, in acute wounds, biofilms can develop in shorter times, and have been found within 14 days, and in some cases within 48 h [17]. Antimicrobial activity against bacteria within biofilms is known to be reduced by 100–1000 times compared to planktonic form. Therefore, a reproducible 3D biofilm model is required to explore and discover efficient antibiofilm approaches.

As a first step toward developing a relevant 3D biofilm model for testing antibiofilm therapies, *S. aureus* SH1000 expressing GFP protein, a standard strain for biofilm related studies, was chosen as a model for this study. This strain has the ability to form a five-fold higher biofilm than other laboratory strains [18]. Planktonic bacteria have previously been used to fabricate beads containing bacteria; however, this might not guarantee achieving biofilm beads. Here, biofilm beads were fabricated utilizing an already-established biofilm that was allowed to grow as a 2D culture for 3 days in humid and low-oxygen conditions. The resultant biofilm clusters within the alginate beads were similar in size and structure to published images of biofilm aggregates in in vivo infections [19]. Analysis of imaging data using a confocal microscope confirmed that the fabricated alginate beads harbored small dense biofilm clusters of bacterial aggregation around 11 µm in size. This is close to the reported diameter of biofilm aggregates in different chronic infections ranging from 5 to 50 μm [19]. It showed the characteristic features of biofilms identified by microscopic investigations of in vivo biofilms detected in various chronic lung infection and wound infections, showing the biofilm phenotype to be aggregated bacteria in clusters embedded within a polymer matrix [19,20].

Though *S. aureus* does not exist in environments of alginate in nature, alginate hydrogels have shown the ability to protect *S. aureus* biofilms from *P. aeruginosa* [21,22]. Alginate has been widely used to provide a matrix in which to embed growing cells, e.g., bacteria and cancer cells, in relatively hypoxic conditions and conditions of low nutrient availability, which is essential to ensure the development of biofilm physiology. Our data also show the ability of alginate to support the establishment of an *S. aureus* biofilm with architecture similar to the in vivo biofilm. 

Gel-based matrices have been utilized as a scaffold for culturing biofilms in vitro, where planktonic bacteria were allowed to grow on the top of gel matrices [23]. However, within this method, biofilm aggregates are directly exposed to the effect of antimicrobials. Conversely, growth of biofilm aggregates within chronic wounds is usually established deep in the wound bed, avoiding direct antimicrobial access. The nature and location of the biofilm in clinical wounds has been found to significantly affect bacterial survival. Bacteriological analysis of surface swabs from chronic wounds showed a decrease in bacterial load after treatment but no significant reduction in bacterial counts from deep tissue biopsies was observed, indicating that microbes at greater depth had enhanced survival [24]. 

Not only do biofilms grow on the surface of chronic wound infections, but they can also colonize deeper tissues and challenge therapeutic remedies [25]. For example, *P. aeruginosa* primarily colonizes the deeper tissues of chronic wounds, though *S. aureus* primarily colonizes near-surface chronic wound tissues. Thus in vitro biofilm models should allow testing of antimicrobials against deep forms of biofilm infection. Various in vitro models have been described in the literature include microtitre plate models, chamber slide model [26,27] Centers for Disease Control reactor models [26,28], drip flow reactor models [29,30], models with gauze [31,32], cellulose filters on agar [33], and Lubbock chronic wound biofilm on agar model [34]. All of these models are designed to test antimicrobials against biofilms that primarily grow on the surface of the model for maximum 96 hrs. 

To mimic the in vivo condition of chronic wounds and overcome the limitations of previous attempts, the fabricated biofilm beads were embedded within two layers of alginate matrices obtaining 3D biofilm model with a thickness 8 mm, which is higher than that previously achieved using bioprinting methods [35]. A unique feature of our model is its design, which deeply embeds biofilms between two thick layers of matrices and allows for in vivo biofilm simulations to test antimicrobials against biofilms growing in deep tissues. In addition, the ability to easily collect and dissolve the matrix for further plating and quantification of live bacterial cells without the need for complex processing steps such as scraping and vigorous spinning is an advantage over other models.

The biofilm growth within the constructed model here was achieved using 3-day-old 2D culture biofilm that was further allowed to mature for 5 days within the beads before combining the beads with the matrix. While most biofilm models utilize biofilms established over 24 h, there is evidence that biofilms mature, reducing susceptibility to antimicrobials with time, suggesting that in vivo biofilms need a longer time to mature. Our 3D model was successful in obtaining mature 8-day-old biofilm, compared to other models which grow biofilm for a maximum of 96 h. The achieved model shares similar characteristics to the clinical setting of biofilm infection in chronic wounds in terms of the structure, maturation, and location of biofilm.

CD and bacitracin are widely used as wound dressings and in topical treatment. However, they fail to completely decontaminate *S. aureus*-infected wounds [36], in addition to their associated cytotoxicity that might interfere with the healing process [37]. We showed that combination of non-antibiotic agents including antiseptics with bacitracin successfully upgraded their capacity to kill planktonic *S. aureus* (although the results are unpublished). Here, bacitracin combined with CD was found be effective against the 2D planktonic *S. aureus* at sub-MIC doses. To compare our constructed 3D biofilm model to 2D culture, 1× and 100× of 6.25 µg/mL bacitracin and 0.000029% CD either alone or combined were applied to the constructed 3D biofilm model. Quantitative analysis was performed through assessing the bacterial load within the matrix, as well as measuring the optical density of the culture medium nourishing the matrix. The results showed that the tested combination at 100× the tested concentration was superior in eradicating the infection, compared to each treatment alone. However, no significant difference was found in the OD of culture medium collected from biofilm models treated with any of the 100× treatments. Single treatments at 100× concentrations prevented dissemination of bacteria from the model into the culture medium, but failed to completely eradicate the biofilm infection. This indicates that bacteria were protected within the matrix away from the antimicrobial effect. This finding was further confirmed by the imaging analysis, revealing that only 100× of the combination was found able to penetrate the biofilm clusters and induce massive bacterial killing.

However, confocal laser scanning imaging (CLSM) showed a slight increase in the PI signals with 1×-bacitracin treatment, which did not align with our quantitative analysis. This may be because PI is a DNA-binding stain which can only cross the cell membrane when it is compromised, and stain the DNA or RNA of the dead cells. Biofilms are composed not only of the bacteria, but also of the extracellular matrix in which they are enclosed, and composed largely of polysaccharides, proteins, and extracellular DNA. Hence, CLSM imaging may reveal a false dead layer of red cells that have green interiors under the red coating layer, indicating that DNA is stained outside of intact membranes [38]. Therefore, this may be the case for 1× bacitracin, implying that qualitative analysis based on fluorescent signals may not be useful in biofilms. Therefore, we validated the viability staining results using CFU counting, which is the most appropriate method as it differentiates between dead and alive bacterial cells. This model makes CFU counting easy.

The result suggests that combinational therapy might be an optimal approach to beat biofilm infections, although some therapies work antagonistically, and combinations of agents need to be chosen carefully. The data also show that the constructed model can mimic the complex in vivo condition, and may ultimately be useful for evaluation and discovery of new anti-biofilm therapies. Suitable antimicrobials can be identified for treatment of chronic wounds using the designed 3D biofilm model within 14–15 days after receiving the sample. This includes 2 days for the isolation of the causative bacteria, 8 days for the manufacture of biofilm beads (including 3 days for establishing the biofilm as 2D culture followed by 3 days of matrix model construction and incubation with tested antimicrobials), and an additional 24–48 h for bacterial quantification. One point is that chronic wounds are not emergencies, and a delay of a few days in starting definitive anti-infection treatment might not be clinically important. In terms of cost, the alginate is very cheap, but Boyden chamber inserts are relatively expensive. However, the overall cost of developing and testing the model is not high, considering the costs of using ineffective antibiotics and the negative consequences for clinical outcomes if conventional susceptibility testing methods are used.

One limitation of this model was the absence of some factors that may interfere with treatment, such as serum and blood. Whether these substances would change the activity of either CD or bacitracin is debatable. CD has been shown to bind to proteins, and to bind to some extent to plasma proteins, but this does not reduce its antimicrobial activity [39]. 

## 5. Conclusions

The current study successfully developed a 3D biofilm model consisting of *S. aureus* biofilm beads encased within two thick layers of alginate matrix. The complex structure of the biofilm identified in confocal images, showing dense clusters of aggregated bacteria, suggests that the 3D model mimics the condition in vivo. Quantitative analysis as well as qualitative imaging data indicated that the generated 3D model may be effective in anti-biofilm susceptibility testing. The current study also demonstrates that combination therapy, at least with these two agents, may be a more effective approach to treating biofilm infections.

## Data Availability

Data are available upon request from the corresponding author.
